# Errors in Text

**DOI:** 10.1001/jamanetworkopen.2021.0724

**Published:** 2021-02-09

In the Invited Commentary “Death by Suicide Among People With Autism: Beyond Zebrafish,”^1^ published January 12, 2021, there were errors in the text. The penultimate sentence of the first paragraph should have read that devastating rates of suicide attempts for autistic girls and women were found. The last sentence of the fifth paragraph should have read that the highest age-based suicide rate in autism was found for individuals aged 30 to 39 years. This article has been corrected.^1^
